# Effect of Potato Starch Hydrogel:Glycerol Monostearate Oleogel Ratio on the Physico-Rheological Properties of Bigels

**DOI:** 10.3390/gels8110694

**Published:** 2022-10-26

**Authors:** Lívia Alves Barroso, Graziele Grossi Bovi Karatay, Miriam Dupas Hubinger

**Affiliations:** Department of Food Engineering and Technology, School of Food Engineering, University of Campinas (UNICAMP), Monteiro Lobato Street, 80, Campinas 13083-862, Brazil

**Keywords:** emulsifier, fat substitute, mixed gel, oil structuring, starch

## Abstract

Bigel (BG) has been shown to be promising for the food industry due to the possibility to manipulate the properties of the system by adjusting the ratio of each individual phase, namely the hydrogel (H) and oleogel (O) phases. This work aimed to evaluate the influence of the O:H ratio on the physical-rheological properties of BG produced with potato starch (PS) and glycerol monostearate (GM). The hydrogel hardness (i.e., 1423.47 g) directly influenced the viscosity of the BG samples, as BG with a higher H-phase presented the highest viscosity and firmness. All BG samples presented shear-thinning behavior and structural breakdown at ~50 °C. BG with a higher O-phase had superior results for thermal stability, softer texture, and yield stress values, representative of good plasticity and spreadability, as compared to BG with less O-phase. The BG with 80% H-phase was less stable during the 21 days of storage in relation to the other BG samples. This study showed the role that the O:H ratio plays in the development of PS-GM-based BGs with tailor-made physical-rheological properties. In addition, the BG is an easily reproduced system with great potential to be used as a *trans* and saturated fat substitute in food applications.

## 1. Introduction

Saturated fatty acids (SFAs) and *trans* fatty acids (TFAs) are commonly used in a broad variety of food products consumed all over the world, such as cookies, cakes, muffins, pizza dough [1,2], chocolate, margarine, cream cheese [3], and meat products [4]. Nonetheless, there are ongoing concerns about the potential adverse health risks associated with consuming these types of fats [5]. In fact, some countries have banned the use of TFAs and suggested a reduction of SFAs from processed foods, as the increase in consumption of foods rich in these fats has been related to the increased propensity for cardiovascular diseases in the world population [6,7,8]. In this context, the search for alternatives to replace SFAs and TFAs with structured unsaturated vegetable oils with similar characteristics to *trans* and saturated fats has been a hot topic of research over the last few years. Among different lipid structures, bigels have demonstrated high potential to be used in a wide variety of food products as fat substitutes [7,9].

Bigel (BG) is a viscoelastic semi-solid structure composed of two phases, namely oleogel (O) and hydrogel (H) [7]. Compared with individual gels (i.e., O and H), BGs have the advantage of having the aforementioned two phases, which enables the combined delivery of hydrophilic and hydrophobic bioactive compounds and the possibility to manipulate the properties of the system by adjusting the ratio of each individual phase [10]. The versatility of BG makes them suitable and interesting for different applications in the cosmetic, pharmaceutical, and food industries [11]. However, despite the promising results, BG has been mainly studied for cosmetic and pharmaceutical applications; and studies are still needed to evaluate and characterize this system for food applications, such as for the replacement of SFAs and TFAs. For such replacement, gel structure and sensory characteristics (i.e., taste and texture) must be maintained as similar to the conventional products as possible in order to facilitate consumers’ acceptance [1].

A wide range of food grade ingredients can be used to produce the H-phase and O-phase. For instance, oleogels produced due to the self-assembly of glycerol monostearate (GM) have been widely studied for food applications as an SFA replacement in chocolate spreads [12], bologna sausage [13], filling creams [14], and baking applications such as in biscuits [15], muffins [16], cakes, and cookies [17]. The use of GM is of interest as self-standing GM oleogels can be produced at concentrations lower than 10% by the direct method [18,19]. Different BGs have been developed using GM as an organogelator [20]. Jiang et al. [21] developed gelatin beads with ethylcellulose oleogels enhanced with GM and observed that GM was mainly responsible for the emulsification and stabilization of water droplets. In the study carried out by Yang et al. [22], BG based on κ-carrageenan hydrogel and oleogel with GM and beeswax were produced, and it was reported that the water/oil holding capacity of the BG was increased with GM content increase.

For the H-phase, a wide range of biopolymers can be used, such as proteins, polysaccharides, and a combination of proteins and polysaccharides [23]. Potato starch (PS) is of great interest as it is a widely consumed and accepted ingredient, is suitable for vegetarian and vegan diets, has good availability and manageability, is low cost, and has high swelling capacity [20,24,25]. Moreover, the PS potential as a thickening agent and gel-hardener makes this starch a promising hydrogelator [26] in the food industry. There are studies reporting the use of wheat starch [27] and pea starch [28] in BG applications. However, to the best of our knowledge, there are no reports of BG using native PS as the hydrogelator in combination with GM as an organogelator.

Studies with BGs aimed at food application are increasing. The study by Martinez et al. [29] reported the production of BGs using candelilla wax (organogelator) and acrylate polymer (hydrogelator). The authors reported that the hydrogelator used seemed to hide oleogel influence over BG in rheological properties. Zheng et al. [30] developed food-grade BG using GM (organogelator) and κ-carrageenan (hydrogelator) and observed that BG could be used as a delivery system for lipophilic bioactive compounds (i.e., β-carotene). Another study produced BG with whey protein concentrate (hydrogelator) and combinations of lecithin and stearic acid and reported the influence of the gelatorson on the functionality and stability of the system [31].

According to Fasolin et al. [32], BG is a system that can be tailored to have specific textures and rheological properties according to the desired application, varying the formulation and process parameters. In this context, this study seeks to evaluate the effects of the O:H ratio variation on the physical and rheological properties, aiming to observe the influence of the individual phases (H and O) on the formation of BGs. Moreover, the study targets the production of a low-cost, stable at room temperature, and easily reproducible BG as a potential replacer for SFAs that could possibly be applied in bakery, meat, chocolate, or spreadable products.

## 2. Results and Discussion

### 2.1. Appearance of Oleogel (O), Hydrogel (H), and Bigels (BGs)

**Figure 1:**
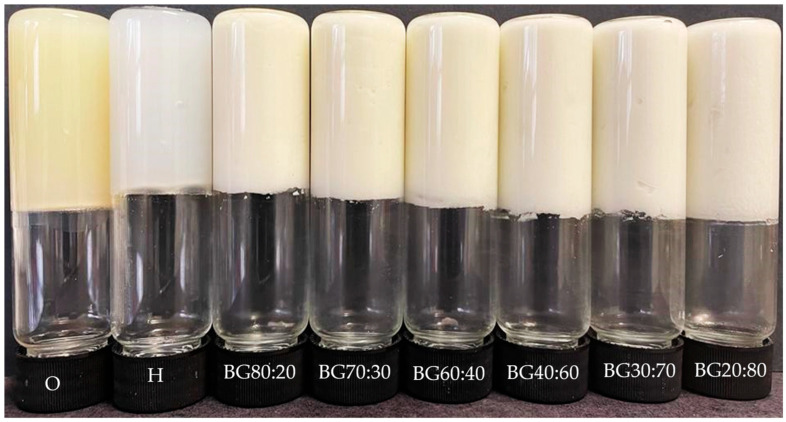
Tube inversion test and the appearance of the oleogel (O), hydrogel (H), and bigel (BG) formulations with different oleogel:hydrogel ratios (O:H).

The ability of a gel to retain its appearance and structural integrity when inverted can be verified by a simple tube inversion test [20]. The tube inversion test, as well as the appearance of the O, H, and BGs with different O:H ratios, can be visualized in Figure 1. The H had an opaque white appearance, while the O had a yellowish appearance. Adding the O-phase to the H-phase altered the appearance of gels to a milky white coloration. The BG samples with a higher content of O-phase had a slightly more yellowish color than samples with a higher content of H-phase. The formation, appearance, and structure of a firm BG system depend on the composition and proportion of both phases. In this study, all BG samples were self-standing after inversion at room temperature, indicating that the concentration of the gelling agents used to form the individual phases and the selected ratios of O:H were suitable for formulating self-supporting BG.

### 2.2. Polarized Light Microscopy

**Figure 2:**
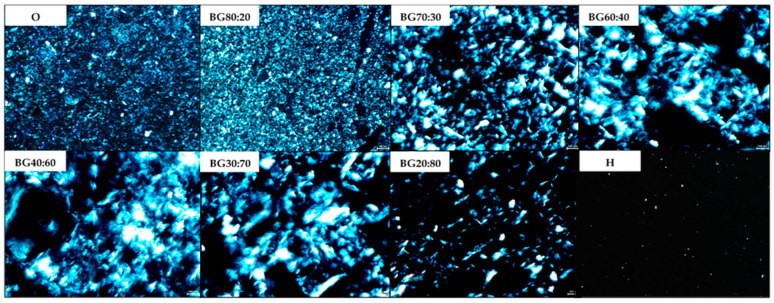
Polarized microscopy images of the hydrogel (H), oleogel (O), and bigel (BG) formulations with different oleogel:hydrogel ratios (O:H).

Optical microscopy was conducted to verify the crystal composition of BGs under polarized light. The polarized light micrographs of the BGs are presented in Figure 2. Fat crystals, represented in this study by the O-phase, are birefringent as they diffract light, making them perform brighter under polarizing microscopy [33]; whereas H are amorphous structures and appear as darker objects under polarizing microscopy [24,25]. Therefore, in this study, the darker points consist of the potato starch (PS) hydrogel while the brighter points consist of the glycerol monostearate (GM) oleogel. Our results agree with those of Larrea-Wachtendorff, Tabilo-Munizaga, and Ferrari [25], who studied PS hydrogel produced by high hydrostatic pressure and observed a darker appearance of the PS hydrogel structure under polarized microscopy. Their study revealed that native potato starch has birefringence; however, the hydrogels produced with PS do not. In their study, the lack of birefringence in the hydrogels produced with PS was related to the gelatinization that occurs during heating to produce the hydrogels. Therefore, it can be assumed that the absence of birefringence indicates the gelatinization of the PS and the formation of the hydrogels.

It can be noted that the higher the O-phase (i.e., BG80:20 > BG70:30 > BG60:40 > BG 40:60 > BG30:70 > BG20:80), the “lighter” the micrographs, as more fat crystals could be observed in the BG network structure. At a higher O-phase concentration (i.e., BG80:20) a higher distribution of fat crystals can be observed in the gel images, which confirms the formation of continuous gel networks. Similar results were found by Samui et al. [34], who also detected a high distribution of GM crystals covering the entire gel image.

In the study by Uslu and Yilmaz [35], who also developed GM oleogels, the presence of small and thin lipid crystals could be observed, which also appeared in the present study for the BG with a higher O-phase (i.e., BG80:20) and in the oleogel micrograph (i.e., O).

Through polarized microscopy, it can be observed that the crystals of the BGs were related to the GM organogelator and PS hydrogelator used to produce the individual phases. It can be concluded that the O-phase influenced the BG even when mixed with a slight H-phase. In addition, the H-phase contributed to the decrease in the birefringence as there was a reduction in the amount of GM crystals.

### 2.3. X-ray Diffraction (XRD)

**Figure 3:**
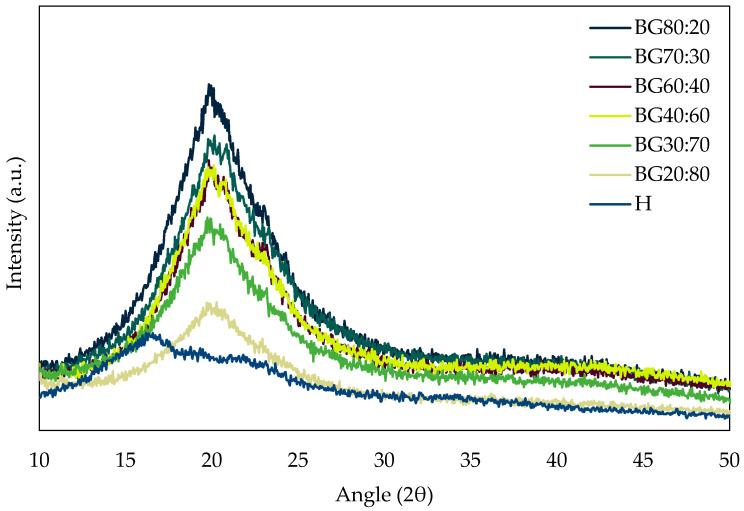
XRD diffractograms of hydrogel (H) and bigel (BG) formulations with different oleogel:hydrogel ratios (O:H).

To better understand the formation mechanism of the BGs, XRD was used to characterize the crystalline structures in the system (Figure 3). The observation of X-ray diffractograms of the BGs showed crystalline peaks at ~20 2θ, while the H-phase did not present a defined peak, hence indicating the major role the O-phase plays in the formation of crystalline structures of the BGs. The increase in the O-phase showed that a structural reorganization occurred, resulting in different molecular packing. Similar peaks were found by Carrillo-Navas et al. [36], who evaluated the GM crystals on water and canola oil interfaces and found sharp peaks between 19.0 and 24.0 2θ.

The absence of a crystalline peak in the H sample is linked to the amorphous nature of potato starch structures, which also influenced the decreases in the peak intensities in BGs with a higher H-phase (i.e., BG20:80). The absence of an apparent peak in the hydrogel diffractogram was also observed by Spagnol et al. [37], who characterized superabsorbent hydrogel nanocomposites based on starch. This confirms that starch hydrogels have a dominant amorphous structure with the absence of intense peaks.

The XRD results are corroborated by the observations from the polarizing micrographs (Figure 2), in which BG with a higher O-phase (i.e., BG80:20) presented higher crystallinity, which is represented in the XRD-diffractogram by the longer and sharper peaks. In contrast, BGs with lower oleogel content (i.e., BG30:70 and BG20:80) presented lower peaks and crystallinity due to more influence of the amorphous H-phase in the system.

According to Sagiri et al. [38], incorporating a higher H ratio hinders the X-ray diffraction of the molecular layers of the GM due to the large and disordered structure of the hydrogel that retains the peaks and their intensity. This was also observed by Ghiasi and Golmakani [27], who developed BG with starch and ethylcellulose at different ratios (25%, 50%, and 75% oleogel fraction). They observed that peak intensities decreased with the H-phase increase. Therefore, it can be observed that the BG structures with a higher O-phase become more ordered systems.

### 2.4. Thermal Properties

**Figure 4:**
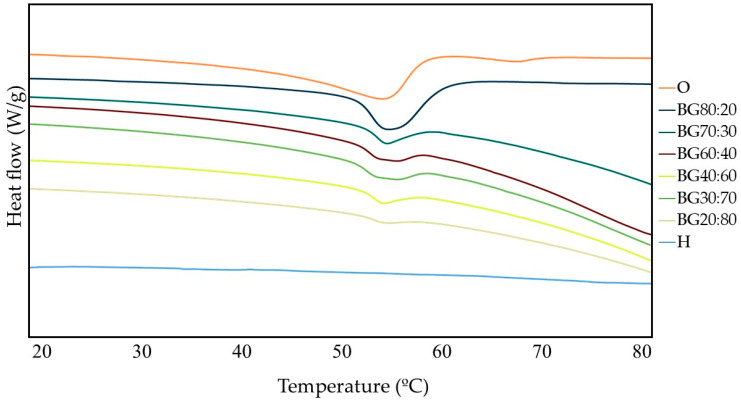
Thermographs of hydrogel (H), oleogel (O), and bigel (BG) formulations with different oleogel:hydrogel ratios (O:H).

A Differential Scanning Calorimetry (DSC) analysis was carried out to characterize the thermal behavior of O, H, and BGs containing different O:H ratios. In Figure 4, melting peak temperatures are depicted, in which BG samples presented a well-defined peak in the heating step in the range of 51.92 °C to 53.70 °C. Even though there was no significant difference (*p* > 0.05) between the BG samples (Table 1), it was observed that as the O-phase increased, the peak became more expressive.

The O-phase showed two melting peaks ranging from 50 to 65 °C, while the H-phase did not show any melting peaks in the evaluated range (Figure 4). Bigels presented endothermic peaks and the influence of O-phase, which were similar to the results found by Zheng et al. [30], who evaluated BG elaborated with GM-based oleogels and κ-carrageenan-based hydrogels in different ratios. In their study, the peak of BG with more O-phase was 55.41 °C, while the BGs with low O-phase were 52.36 °C.

Zhu et al. [39] who evaluated BGs with different GM and gellan gum hydrogel ratios found lower endothermic peaks in the BGs (49.8–50.2 °C); and as in our results, no melting peak was observed for the H-phase, indicating that the range evaluated in the present study was lower than the temperature of evaporation of free water.

The enthalpy was found to be higher in BGs containing a higher O-phase concentration (Table 1); this can be related to the crystallinity of the GM oleogel presented in BG [40], which was also consistent with the results obtained in XRD.

Therefore, the difference observed in the melting temperatures of O and BG suggests the influence of the H-phase on decreasing the enthalpy. Probably the reason is that the enthalpy was proportional to the crystal content in the structure, so systems with low or no crystallinity should have the enthalpy of melting close to zero [41].

### 2.5. Rheological Properties

#### 2.5.1. Apparent Viscosity

**Figure 5:**
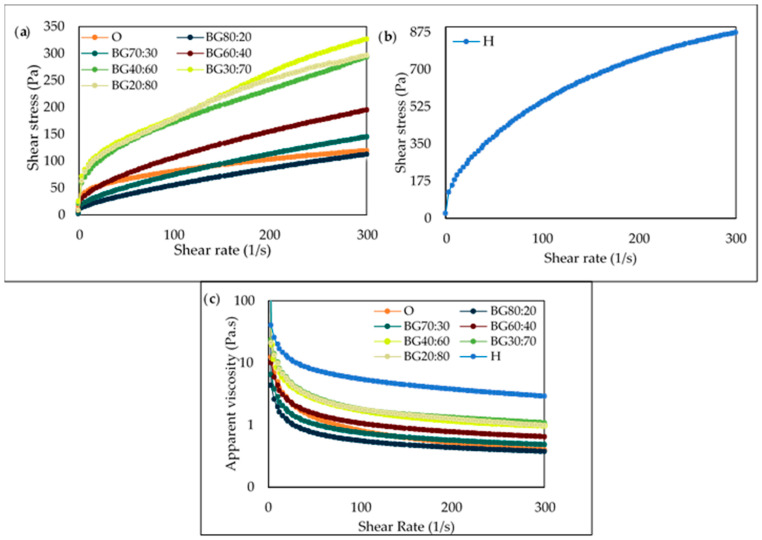
Shear stress *versus* shear rate of oleogel (O) and bigel (BG) formulations with different oleogel:hydrogel ratios (O:H) (**a**), shear stress *versus* shear rate of the hydrogel (H) (**b**), and apparent viscosity of hydrogel (H), oleogel (O), and bigel (BG) formulations with different oleogel:hydrogel ratios (O:H) (**c**).

The shear rate *versus* the shear stress of O, H, and BG samples can be visualized in Figure 5a,b. It can be noted that all samples presented a similar initial shear stress upon the application of a shear rate. In Figure 5c, it is possible to observe that the apparent viscosity of all samples decreased under increasing shear rates, evidencing a shear-thinning behavior, which is typical of non-Newtonian fluids.

The apparent viscosity of the different formulations at two different shear rates, namely 3 s^−1^ (ƞ3) and 300 s^−1^ (ƞ300), are shown in Table 2. The lowest apparent viscosity was observed for the BG with a high O-phase (i.e., BG80:20), while the highest apparent viscosity was observed for the BG with a higher H-phase (i.e., BG20:80). This behavior can be directly related to the apparent viscosity of the individual phases, as the highest apparent viscosity was for the H-phase (i.e., H) and the lowest was for the O-phase (i.e., O). Therefore, it is possible to produce BGs with the desired viscosity simply by adjusting the O:H ratio. In other words, when a more viscous BG is desired, a higher amount of H-phase should be added. Yet, it is important to note that there was no statistically significant difference between all BG and O samples at the shear rate of 300 s^−1^. These results indicate that when incorporated into the BG matrix, the effect of H on the apparent viscosity is not so pronounced. Therefore, it can be assumed that the use of lower concentrations (i.e., <8%) could also be of interest, aiming at the use of fewer ingredients, while still maintaining the possibility of obtaining BG with tunable viscosity properties. In other words, this could be done not only by adjusting the O:H ratio but possibly by also reducing the concentration of the H-phase.

Moreover, the concentration of the potato starch polymer used to produce the H-phase contributed to a great extent in promoting a highly viscous gelatinized hydrogel phase. The H-phase was a more resistant gel (i.e., 870 Pa), in comparison to the BG samples, which presented values from 70 Pa (BG80:20) to 300 Pa (BG20:80).

The modeled parameters, namely the flow consistency index (k) and the flow behavior index (*n*), can be seen in Table 2. For k, there were significant differences (*p* < 0.05) among the BG samples. The k parameter is related to the apparent viscosity and the higher the k value, the higher the viscosity is [42,43]; this confirms the apparent viscosity results, which suggest that from BG40:60 (25.44 ± 6.20) to BG20:80 (27.86 ± 2.90), the BG samples were more structured and firmer in comparison to the other BG samples and did not present statistical differences (*p* > 0.05). As for n, all the BG samples presented *n* < 1, which corresponds to a pseudoplastic behavior [44,45]. It is worth mentioning that BGs with a higher H-phase presented higher *n* values, suggesting a more structured gel when compared to gels containing lower H-phase content.

Therefore, BGs tended to have different gel strengths than the corresponding hydrogel and oleogel, implying the occurrence of an associated effect by mixing the H and O-phases. This effect could be ascribed to the interpenetration of the O and H networks [44].

These results are promising as the variation in the O:H ratio is a relevant factor in enabling the control of the rheological behavior of the BG systems, as it allows the production of BG with tailor-made characteristics.

#### 2.5.2. Amplitude Sweep

**Figure 6:**
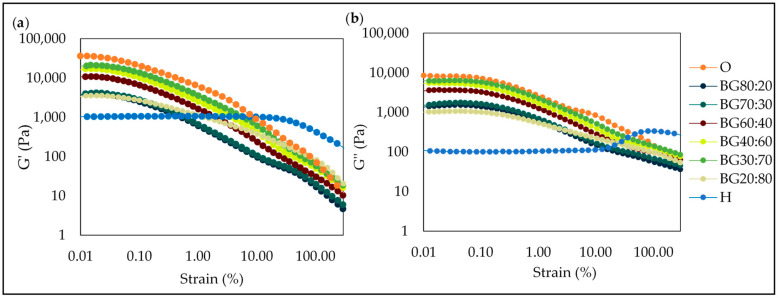
Amplitude sweeps for oleogel (O), hydrogel (H), and bigel (BG) formulations with different oleogel:hydrogel ratios (O:H) at 25 °C: The storage modulus (G′) (**a**) and the loss modulus (G″) (**b**).

The amplitude sweep allows for the identification of the linear viscoelastic region (LVR), which corresponds to a region where the sample does not show a structural breakdown under the application of mechanical forces [46]. Figure 6 shows the amplitude sweeps of all BG and individual gels (O and H). Results showed that G′ was higher than G″ within LVR, indicating a predominantly elastic behavior. BG was formed in all ratios evaluated in this study, and the LVR was similar for all BG samples.

The value at which G′ sharply decreases is defined as the critical oscillatory stress or strain, also known as the limiting value of oscillatory strain (OSL). The OSL determines the maximum deformation that a system can withstand without structural breakdown [47,48]. In this study, the H had the highest OSL (i.e., 21.70%). This outcome indicates that the higher H-phase concentration improves the strength and rigidity of the system as the OSL of the BG containing a higher H-phase also presented higher values of OSL (i.e., 0.04–0.05%) as compared to the O-phase (i.e., 0.02%). Above these OSL values, the storage modulus (G′) showed pronounced decay in the BG sample. At this stage, a crossover point (G′ = G″) occurred, indicating a breakdown of the structural network of the BG samples. Rheological measurements from this study showed similar variation behavior as compared to other studies carried out with BG containing GM oleogel. As reported by Jiang et al. [21], the influence of the O-phase in the lowest strain can be related to the viscoelasticity of GM oleogel; which in amplitude sweep was significantly weakened from shearing.

#### 2.5.3. Frequency Sweep Test

**Figure 7:**
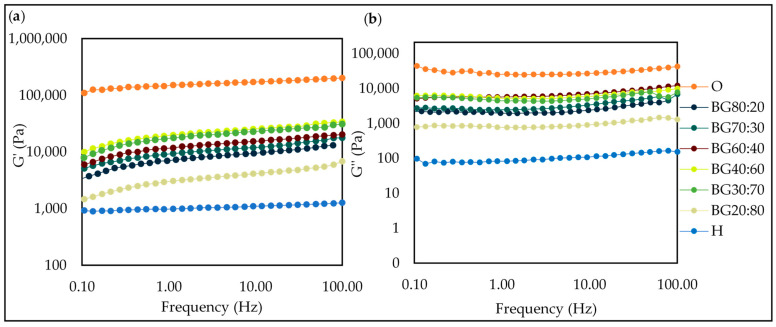
Frequency sweeps for oleogel (O), hydrogel (H), and bigel (BG) formulations with different oleogel:hydrogel ratios (O:H) at 25 °C: The storage modulus (G′) (**a**) and the loss modulus (G″) (**b**).

The G′ and G″ of all BGs remained nearly unchanged and did not cross, indicating that the BG samples were independent of the applied frequency in the tested experimental range of 0.01–100 Hz and demonstrating the presence of structured systems at different frequencies. All the samples showed a predominance of the storage modulus (G′, representing the elastic property) over the loss modulus (G″, depicting the viscous property), indicating more gel-like behavior. In Figure 7, it can be observed that the BG samples were between O-phase (highest—G′ and G″) and H-phase (lowest—G′ and G″).

Similar results were found by Zheng et al. [30] who reported that G′ of the BGs with GM and κ-carrageen was much larger than G″, indicating that typical elastic gel networks had been formed.

The GM oleogel used to produce the O-phase was shown to promote a firm gel with a high G′ value (>10^5^ Pa), as presented in Figure 7, while the PS hydrogel used showed a smaller value of the elastic property (G′). With respect to the BGs, it was observed that there was a decrease in the elastic property of the oleogel when combined with the H-phase. All the BG samples presented a solid-like behavior, whereas the BG40:60 and BG30:70 presented the highest G′, which meant that they had a more pronounced solid-like behavior as compared to the other samples. This finding confirmed that the BG can offer higher gel strength than pure hydrogel as a fat replacer.

#### 2.5.4. Temperature Ramp Test

**Figure 8:**
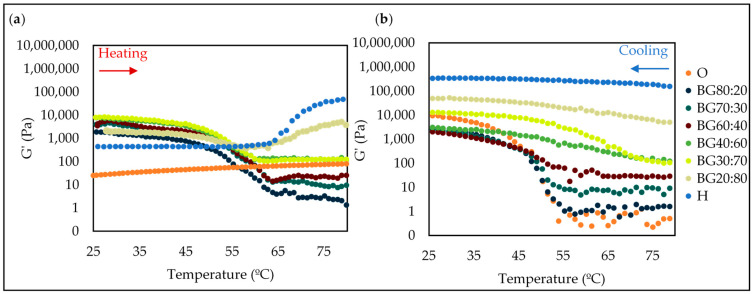
Temperature sweeps for oleogel (O), hydrogel (H), and bigel (BG) formulations with different oleogel:hydrogel ratios (O:H): heating(**a**) and cooling steps (**b**).

Thermodynamic properties of the BGs developed with the individual phases were evaluated through a temperature sweep test (Figure 8) to measure the system resistance to the applied temperature. 

At the earlier stage of heating (5–45 °C), storage modulus (G′) of the BGs with a higher O-phase (i.e., BG80:20, BG70:30, BG60:40, BG40:60, and BG30:70) was almost constant (Figure 8), and after a structure breakdown at ~50 °C, a fast decrease in storage modulus was observed, suggesting the onset of destabilization of the systems. The most pronounced G′ decrease occurred at 50–65 °C, which corresponded to the phase transition temperatures of GM oleogels (melting of GM) [49]. At temperatures above 60 °C, most BGs had higher G′ than G″, indicating that the systems had more elastic than viscous properties.

At this temperature range, only BG20:80 presented a stable system, while all the others showed a typical abrupt phase transition at 60 °C, demonstrating the possible application limit of this system (Figure 8). This abrupt phase transition was also observed by Habibi et al. [50], who evaluated the effects of GM oleogel, κ-carrageenan hydrogel, and whey protein hydrogel on the properties of BGs, finding the temperature zone of decrease between 50 and 63.3 °C.

Due to the thermal reversibility of GM [35], the O-phase was stable during the entire range of heating. BGs with a high H-phase presented higher temperature stability with a more delayed abrupt phase transition. This result suggests the G′ of the BGs was greatly affected by temperature and the melting of GM crystals and the temperature resistance of PS hydrogel; a similar finding was also reported by Lupi et al. [51], that produced BGs samples with rice bran wax and GM as organogel, and BGs with different oleogel/hydrogel ratios.

### 2.6. Mechanical Properties

Based on the results of Table 3, all BG samples provided systems that showed semi-solid textures. The BG capacity to provide semi-solid consistency is an important attribute, which contributes to the assurance of good spreadability of the material. The firmness represents the maximum compression force to deform the structure by a certain distance [15]. In the present study, statistically significant differences (*p* < 0.05) in firmness were found among the individual phases (H and O), while no significant difference (*p* > 0.05) was found between O and BG with BG80:20 to BG30:70.

However, the BG20:80 sample with a higher H-phase had the most rigid structure (i.e., 371.00 g) when compared to the other BGs with a lower H-phase (i.e., BG80:20), which had a firmness of 99.77 g. Thus, the BGs with higher H-phase were more susceptible to resisting an applied mechanical force.

It was also observed that GM oleogel influenced the firmness of the other BG samples. The firmness parameter indicates, to some extent, the ease at which a semi-solid product can be spread; this parameter is related to the yield value. According to Haighton [52], the yield value can be used for the classification of fatty products. Yield values from 200 to 800 present characteristics of good plasticity and spreadability of a system. In this study, the BG samples from BG80:20 to 30:70 fall into this range, though BG20:80 can be classified as stiff but satisfyingly spreadable. Regarding the oleogel, the present structure is characteristic of a soft and spreadable gel, while the hydrogel showed values classified as very hard.

Cohesiveness corresponds to the strength of internal structure bonds [53], and is directly related to hardness and fracture [54]. Our study observed a decrease in cohesiveness in BG samples with a higher O-phase. These results are corroborated by the rheological measurements, in which the lowest apparent viscosity was observed for the BG with a high O-phase (i.e., BG80:20). The harder the material, the more cohesive it is; in the present study, as H-phase had a harder structure, the BG samples with more H-phase presented higher cohesiveness. Golodnizky et al. [55] and Habibi et al. [50,56] reported that the individual phases and their biopolymer concentration and type influenced the mechanical properties of bigels, as was also observed in our study. It is worth mentioning that a direct comparison among studies is not that straightforward as the O:H ratio is not the only parameter that should be taken into account when analyzing the cohesiveness results. The type of hydro and oleogelator used as well as their concentration also play a major role in texture and rheological parameters.

Concerning the stickiness, independent of the ratio, the BG samples had no significant difference (*p* > 0.05), while the O-phase and H-phase presented differences among each other. For all the five texture parameters evaluated by the compression method, the values were found to be the highest in the hydrogel sample (stickiness and adhesiveness were negative, but their values were higher). Moreover, the high proportion of H-phase in the structure increased firmness and other parameters in the bigel sample, while the O-phase increase did not present a difference statistically (*p* > 0.05) among the samples (i.e., BG80:20 and BG70:30). This result agrees with the rheology data, in which the H sample was the most viscous and strong when compared to the O and BG samples. These results emphasize the potential of the variation of the O:H ratio to form systems with tailored textures. These systems may be used to form more sustainable, low-cost, reproducible SFA and TFA substitutes.

### 2.7. Oil Loss (OL)

**Figure 9:**
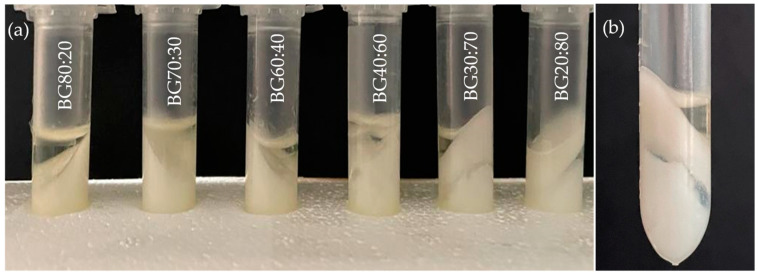
Appearance of bigel samples after centrifugation: Bigel formulations with different oleogel:hydrogel ratios (O:H) upon centrifugation (**a**) and the different layer formation (**b**).

After the centrifugation, different phases were observed (Figure 9). The formation of a top layer in the Eppendorf^®^ made it unfeasible to calculate the oil loss accurately (Figure 9b), as the oil phase was located below this layer. 

The bigel showed low oil holding ability at 5 °C, with an apparent higher oil release in bigels with a higher O-phase (Figure 9a).

Reports from the literature state that oleogels produced with GM are efficient oil loss reducers since the high crystalline and tiny crystals of the GM (Figure 2) cause them to reduce the number of pores and increase the available area to reduce OL [56]; in this way, the crystal lattice physically traps the oil. However, when in combination with the H, the GM oleogel did not seem to be such an efficient oil reducer as a high amount of oil release was observed. Therefore, the bigel with more O-phase presented more oil loss than with more H-phase, and it was visually observed that BGs with an intermediate ratio and more H-phase showed better results in oil loss, i.e., BG60:40, BG40:60, and BG30:70.

### 2.8. Physical Stability Measurements

**Figure 10:**
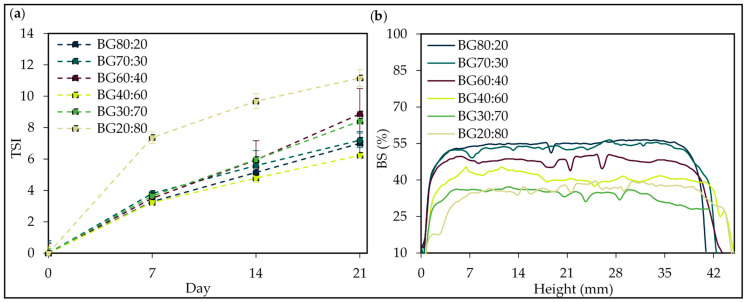
Turbiscan stability index (TSI) (**a**) and backscattering profiles (BS) (**b**) over 21 days of storage of bigel formulations with different oleogel:hydrogel ratios (O:H) at 25  °C.

To evaluate the physical stability of bigels, dynamic light scattering [57] measurements were carried out. The kinetic profiles throughout the 21 days of storage are shown in Figure 10.

The Turbiscan stability index (TSI) results were higher for the BG20:80 sample, revealing a level of instability varying from 0.0 ± 0.0 to 11.2 ± 0.5; since the higher the TSI value, the more unstable the system becomes [58]. In this context, the bigels with better stability were BG40:60, BG80:20, and BG70:30, which varied from 0 to 5.9 ± 0.1, 6.3 ± 0.9 and 6.8 ± 0.5, respectively.

Turbiscan backscatter data were plotted against sample height over time, and the deflocculation/coagulated emulsion stability was evaluated by analyzing the backscatter fingerprints obtained from Turbiscan [59]. The transmission and backscattering profiles (BS) of BGs (Figure 10b) were close to the baseline curve during the 21 days of storage; samples of bigels with a higher O-phase (i.e., BG80:20 and BG70:30) showed a higher BS value (~55 %).

These results provide evidence of the homogeneous opalescent aspect of all BGs and the absence of any segregation phenomenon, as further demonstrated by the stability kinetics profiles in Figure 10b. The decrease in the BS intensity in the BG can be attributed to the increase of the H-phase; as water has a higher density than oil, the water present in the samples has glided to the bottom, which leads to sedimentation and an increase in BS [58]. Therefore, samples with a higher O-phase presented good stability.

## 3. Conclusions

The current study developed a novel biphasic viscoelastic system, namely food-grade bigel, using potato starch as a hydrogelator and glycerol monostearate as the organogelator. The research revealed that the structural properties of the bigels were significantly affected by the oleogel and hydrogel ratio. The increase in the O-phase resulted in enhanced thermal stability, softer texture, more crystalline system, lower viscosity, and good plasticity and spreadability from yield stress values of bigel.

All samples under study were non-Newtonian shear thinning fluids and semi-solid systems. The strength of gels was directly dependent on the O:H ratio, in which the H-phase significantly increased the viscosity and strength of the bigels. Results showed that the bigels can be developed aimed at developing tailor-made characteristics. For instance, if the aim is to have firmer gels, a higher amount of H-phase should be added, whereas if the aim is to have more spreadable gels, a higher O-phase should be added.

## 4. Materials and Methods

### 4.1. Materials

Potato starch (PS) powder (C Gel™ LM 30,008, 91% carbohydrates, 0.4% of protein, 0.3% of ash, 0.1% of fat, and 8.2% of moisture) and soybean oil without antioxidants were kindly donated by Cargill (Mairinque, Brazil). Glycerol monostearate (GM) (>95.0% purity) was purchased from AlfaAesar^®^ (Ward Hill, MA, USA). Sodium azide was obtained from Sigma-Aldrich (St. Louis, MI, USA), and all other chemical reagents were of analytical grade.

### 4.2. Bigel (Oleogel-in-Hydrogel) Preparation

*Hydrogel preparation*: A gelation test with various concentrations (i.e., from 1% to 10%) was carried out to verify and define the concentration to be used for the hydrogel production (Figures S1 and S2). Based on these results, a concentration of 8% (*w*/*w*) of potato starch (PS) was chosen as the hydrogelator to produce the hydrogel (H-phase). The PS was dispersed in deionized water containing 0.1% of sodium azide under continuous magnetic stirring (300 rpm) for 10 min at 85 °C. After that, the sample was kept in a biochemical oxygen demand (BOD) incubator for 24 h at 25 °C to ensure the complete gelation of the H-phase [27].

*Oleogel preparation*: The concentration of 8% (*w*/*w*) of glycerol monostearate (GM) was chosen as the organogelator to produce the oleogel (O-phase). The GM was mixed with 92% (*w*/*w*) of soybean oil under continuous magnetic stirring (500 rpm) for 30 min at 90 °C. The temperature of 90 °C was selected to ensure melting and erasing of the crystal memory of the GM. After that, the sample was kept in a BOD incubator for 24 h at 25 °C to ensure O-phase complete gelation [32].

*Bigel preparation*: To verify the influence of individual phase concentration on the appearance, structure, thermal, and strength of the bigel a variation of the oleogel:hydrogel ratio (O:H) was carried out. The tested O:H ratio consisted of 80:20, 70:30, 60:40, 40:60, 30:70, and 20:80, and the BG samples were named as BG80:20, BG70:30, BG60:40, BG40:60, BG30:70, and BG20:80, respectively. After the preparation and structuring of the individual phases (i.e., 24 h at 25 °C in BOD as described above), the O-phase was mixed with the H-phase using a rotor-stator device (Ultraturrax^®^ T18 basic, IKA^®^-Werke GmbH & Co., KG, Staufen, Germany) equipped with a disperser (S18N, IKA^®^-Werke GmbH & Co., KG, Staufen, Germany) operating at 10.000 rpm for 3 min for the formation of a white and homogeneous bigel [39].The bigels were stored at a temperature of 25 °C for 24 h in a BOD incubator for the structuring and formation of bigels.

### 4.3. Polarized Microscopy Analysis

The crystal morphology of BGs and individual gels (O and H) was investigated using a Polarized Light Microscope (Olympus System Microscope model BX 50, Olympus America Inc., Center Valley, PA, USA) equipped with a digital camera (Nikon DS-Ri1, Melville, NY, USA). Images were analyzed with NIS-Elements Microscope Image Software (Nikon, Melville, NY, USA). A drop of bigel was placed on a glass slide, gently covered with a cover slip, and analyzed at room temperature.

### 4.4. X-ray Diffraction (XRD)

The crystallinity of the bigels was determined according to the American Oil Chemists Society (AOCS) Cj 2–95 method [60]. Previously, the samples (H and BG) were freeze dried for 48 h prior to analysis. The H and BG samples were read in an X-ray diffractometer (Philips, PW 1710, PANalytical, Almelo, Netherlands) using Bragg–Brentano geometry (θ:2θ) with Cu-Kα radiation (λ = 1.54056 Å) at 40 kV, 40 mA. The scanning 2θ range of the samples was 5−50° and the scanning interval was 0.05° 2θ/s.

### 4.5. Thermal Properties

A differential scanning calorimeter (Model 2920, TA Instruments, New Castle, NH, USA) was used to analyze the thermal properties of the bigels. The bigel samples (0–15 mg) were sealed in aluminum pans, and an empty aluminum pan without a sample was set as a reference. The sealed pans were taken to the equipment for analysis. The thermal profile was determined in the heating temperature range from −40 °C to 80 °C at a rate of 5 °C/min, under an inert nitrogen atmosphere at a flow rate of 10 mL/min [61]. All sample measurements were done in triplicate.

### 4.6. Rheological Measurements

#### 4.6.1. Apparent Viscosity

The apparent viscosity was evaluated using a Physica MCR 301 stress-controlled rheometer (Anton Paar, Graz, Austria) with parallel-plate geometry (Ø = 50 mm, type PP50/S and 1 mm gap) by performing flow curves with shear rate values ranging from 0.1 to 300 s^−1^ at 25 °C. For each sample, at least three replications were performed.

#### 4.6.2. Dynamic Rheological Measurements

The dynamic rheological properties of the bigels were carried out using a stress-controlled rheometer (Physica MCR 301, Anton Paar, Graz, Austria) coupled with parallel-plate geometry (Ø = 50 mm, type PP50/S and 1 mm gap) and a Peltier system (Viscotherm VT2, Phar Physica) (±0.1 °C). First, an amplitude sweep test was conducted over the shear strain range of 0.01–100% and fixed 1 Hz frequency to define the linear viscoelastic region (LVR). A frequency sweep test was then performed in the LVR region by increasing angular frequency from 0.1 to 100 Hz at a fixed shear strain of 0.01% at room temperature (~25 °C). The behavior of the storage (G′) and loss (G″) modules was obtained as a function of frequency. A temperature ramp test was performed by heating the samples from 25 to 90 °C with an up and down ramp (25 °C/90 °C) at a rate of 5 °C/min and a frequency of 1 Hz. For each sample, at least three replications were performed.

### 4.7. Mechanical Properties

The mechanical properties of the bigels were evaluated using a texture analyzer (TA-TX Plus, Stable Micro Systems, Surrey, UK). The firmness, compressibility, and adhesiveness of the samples were determined from the stress-strain curves using the instrument software Exponent Connect. For measurements, samples were put in a 50 mL cylindrical glass beaker and compressed using a probe (AP/45C) with a pre-test velocity of 8.0 mm/s, followed by a test velocity, and a post-test velocity of 10.0 mm/s, with a force of 10.0 g. The probe was immersed in the bigel to a depth of 3.0 cm and then returned to the starting position [39]. For each sample, at least five replications were performed.

### 4.8. Oil Loss (OL)

The oil loss (OL) of bigels was measured qualitatively under centrifugal forces as in the study by Mao et al. [62]. About 1 g of each sample of different O:H ratios was poured into an Eppendorf. Samples were centrifuged at 8600 g for 30 min at 5 °C in a microtube centrifuge (Eppendorf^®^, Model 5418 R, São Paulo, Brazil).

### 4.9. Physical Stability Measurements

The physical stability of the bigels (approximately 20 mL) was verified in terms of phase separation (destabilization) and visible discolorations during 0, 7, 14, and 21 days of storage. The phase separation equilibrium of bigels was evaluated, by a vertical scan analyzer, based on an optical scanning spectrophotometer equipment (Turbiscan LAB^®^ Expert, MA 2000, Formulation, L’Union, France), coupled with TurbiSoft software. 2.0.0.28. The light source passes through the glass tube containing the sample, and two sensors measure the amount of transmitted light: transmission (TSI)—which represents clarification, and the amount of scattered light: backscattering (E)—which represents sedimentation. All sample measurements were done in triplicate at 25 °C.

### 4.10. Statistical Analysis

The results were presented as mean ± standard deviation. Statistical differences between treatments were evaluated by analysis of variance (ANOVA) and Tukey tests (*p* < 0.05) using Statistica software version 10.0 (Statsoft^®^, Tulsa, OK, USA).

## Figures and Tables

**Table 1:** Thermal parameters of the hydrogel (H), oleogel (O), and bigel (BG) formulations with different oleogel:hydrogel ratios (O:H).

Sample	T_onset_ (°C)	T_peak_ (°C)	∆H (J/°C)
O	45.74 ^b^ ± 0.25	53.11 ^a^ ± 0.03	4.22 ^a^ ± 0.76
BG80:20	50.61 ^a^ ± 0.25	53.70 ^a^ ± 0.43	2.23 ^b^ ± 0.29
BG70:30	50.14 ^a^ ± 0.36	52.95 ^a^ ± 0.84	1.93 ^bc^ ± 0.36
BG60:40	50.57 ^a^ ± 0.12	52.27 ^a^ ± 0.52	2.09 ^bc^ ± 0.02
BG40:60	50.45 ^a^ ± 0.48	51.92 ^a^ ± 0.21	1.24 ^bc^ ± 0.83
BG30:70	49.22 ^a^ ± 1.69	53.25 ^a^ ± 2.35	1.51 ^bc^ ± 0.07
BG20:80	50.16 ^a^ ± 0.49	53.20 ^a^ ± 1.76	0.37 ^c^ ± 0.17
H	-	-	-

The results (mean value ± standard derivation) with different letters in lower-case superscripts in the same column indicate a significant difference between the formulations (*p* < 0.05) by the Tukey’s test.

**Table 2:** Apparent viscosity (ƞ) at a shear rate of 3 (ƞ_3_) and 300 (ƞ_300_) s^−1^ measured at 25 °C and flow consistency (k) and flow behavior (*n*) index obtained from the power law model with its corresponding coefficient of determination (R^2^) of the hydrogel (H), oleogel (O), and bigel (BG) formulations with different oleogel:hydrogel ratios (O:H).

Sample	ƞ_3_	ƞ_300_	k	*n*	R^2^
O	12.30 ^bd^ ± 1.75	0.99 ^b^ ± 1.02	20.02 ^b^ ± 2.86	0.79 ^c^ ± 0.00	0.98
BG80:20	4.36 ^d^ ± 1.79	0.38 ^b^ ± 0.09	3.47 ^c^ ± 1.85	0.38 ^b^ ± 0.00	0.99
BG70:30	6.51 ^d^ ± 2.03	0.48 ^b^ ± 0.07	5.68 ^c^ ± 2.33	0.48 ^b^ ± 0.00	0.99
BG60:40	10.10 ^d^ ± 3.39	0.65 ^b^ ± 0.07	10. 48 ^c^ ± 4.29	0.64 ^bc^ ± 0.00	0.99
BG40:60	19.63 ^bc^ ± 5.35	0.98 ^b^ ± 0.17	25.44 ^b^ ± 6.20	0.70 ^c^ ± 0.01	0.98
BG30:70	21.00 ^c^ ± 6.20	1.09 ^b^ ± 0.23	23.37 ^b^ ± 5.99	0.69 ^c^ ± 0.00	0.97
BG20:80	23.60 ^c^ ± 1.59	0.99 ^b^ ± 0.07	27.86 ^b^ ± 2.90	0.79 ^c^ ± 0.00	0.98
H	30.60 ^a^ ± 0.59	1.41 ^a^ ± 0.07	47.74 ^a^ ± 0.58	0.99 ^a^ ± 0.00	0.99

The results (mean value ± standard derivation) with different letters of lower-case superscripts in the same column indicate a significant difference between the formulations (*p* < 0.05) by the Tukey’s test.

**Table 3:** Textural properties of oleogel (O), hydrogel (H), and bigel (BG) formulations with different oleogel:hydrogel ratios (O:H).

Sample	Yield Value (gF/cm²)	Firmness (g)	Cohesiveness (g.s)	Stickiness (g)	Adhesiveness (g.s)
O	144.77 ^c^ ± 14.35	144.77 ^c^ ± 3.83	123.33 ^c^ ± 7.57	−233.87 ^b^ ± 13.34	−49.33 ^b^ ± 9.61
BG80:20	295.86 ^c^ ± 1.97	99.77 ^c^ ± 0.67	104.34 ^c^ ± 8.50	−44.07 ^c^ ± 4.36	−30.33 ^b^ ± 0.58
BG70:30	299.12 ^c^ ± 2.59	100.85 ^c^ ± 0.87	159.65 ^bc^ ± 2.79	−48.77 ^c^ ± 1.21	−30.33 ^b^ ± 4.04
BG60:40	299.07 ^c^ ± 0.71	100.87 ^c^ ± 0.24	188.45 ^bc^ ± 8.35	−55.75 ^c^ ± 1.69	−72.75 ^ab^ ± 11.96
BG40:60	312.17 ^c^ ± 1.68	105.27 ^c^ ± 1.53	244.97 ^bc^ ± 16.26	−57.80 ^c^ ± 1.93	−124.67 ^a^ ± 8.14
BG30:70	403.51 ^c^ ± 5.4	136.07 ^c^ ± 7.09	275.00 ^bc^ ± 4.00	−55.53 ^c^ ± 1.11	−130.75 ^a^ ± 1.29
BG20:80	1100.30 ^b^ ± 9.40	371.00 ^b^ ± 9.40	453.00 ^b^ ± 26.67	−71.03 ^c^ ± 1.12	−87.67 ^ab^ ± 10.69
H	3221.290 ^a^ ± 28.71	1423.47 ^a^ ± 3.35	1534.00 ^a^ ± 20.10	−1200.03 ^a^ ± 65.93	−37.67 ^b^ ± 2.89

The results (mean value ± standard derivation) with different letters of lower-case superscripts in the same column indicate a significant difference between the formulations (*p* < 0.05) by the Tukey’s test.

## Data Availability

Raw data on the manuscript is public.

## Supplementary Materials

The following supporting information can be downloaded at: https://www.mdpi.com/article/10.3390/gels8110694/s1, Figure S1: Tube inversion test with various concentrations (i.e., 1% to 10%) of potato starch hydrogel. Figure S2: Potato starch hydrogel with various concentrations (i.e., 1% to 10%). Figure S3: Appearance of bigel (BG) formulations with different oleogel:hydrogel ratios (O:H) after 21 days of storage at 25 °C.
