# Supplementary material for: Effect of Potato Starch Hydrogel:Glycerol Monostearate Oleogel Ratio on the Physico-Rheological Properties of Bigels
Source: Gels. 2022 Oct 26;8(11):694. doi: 10.3390/gels8110694 (PMC9689572; doi:10.3390/gels8110694)
Supplement: Supplementary file 1 [file gels-08-00694-s001.zip › gels-1986333-supplementary.pdf]

Supplementary Materials

Effect of Potato Starch Hydrogel: Glycerol Monostearate Oleogel Ratio on the Physico-Rheological Properties of Bigels

Lívia Alves Barroso *, Graziele Grossi Bovi Karatay and Miriam Dupas Hubinger

Department of Food Engineering and Technology, School of Food Engineering, University of Campinas (UNICAMP), Monteiro Lobato Street, 80, Campinas 13083-862, Brazil
* Correspondence: livia.barroso@hotmail.com

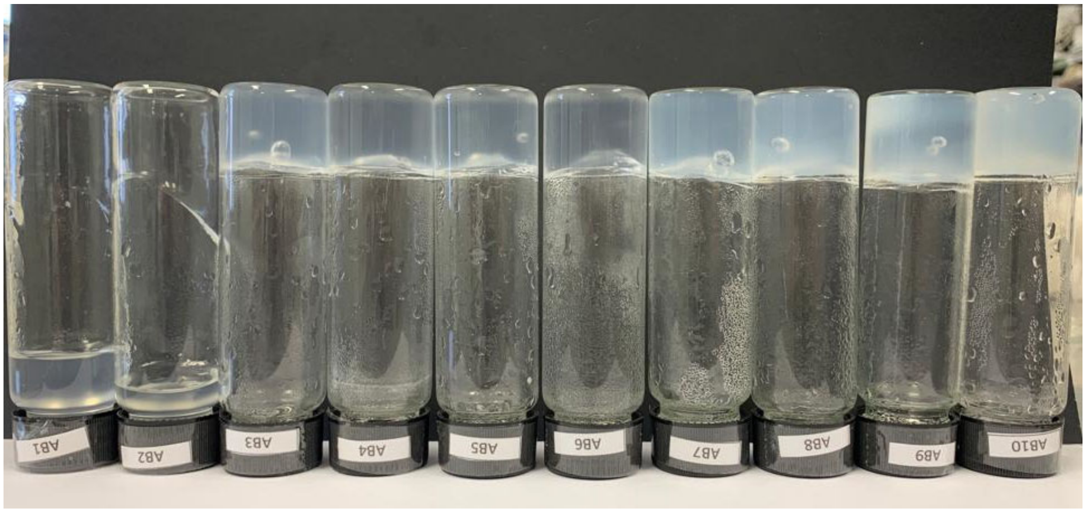

Figure S1. Tube inversion test with various concentrations (i.e., 1% to 10%) of potato starchhydrogel.

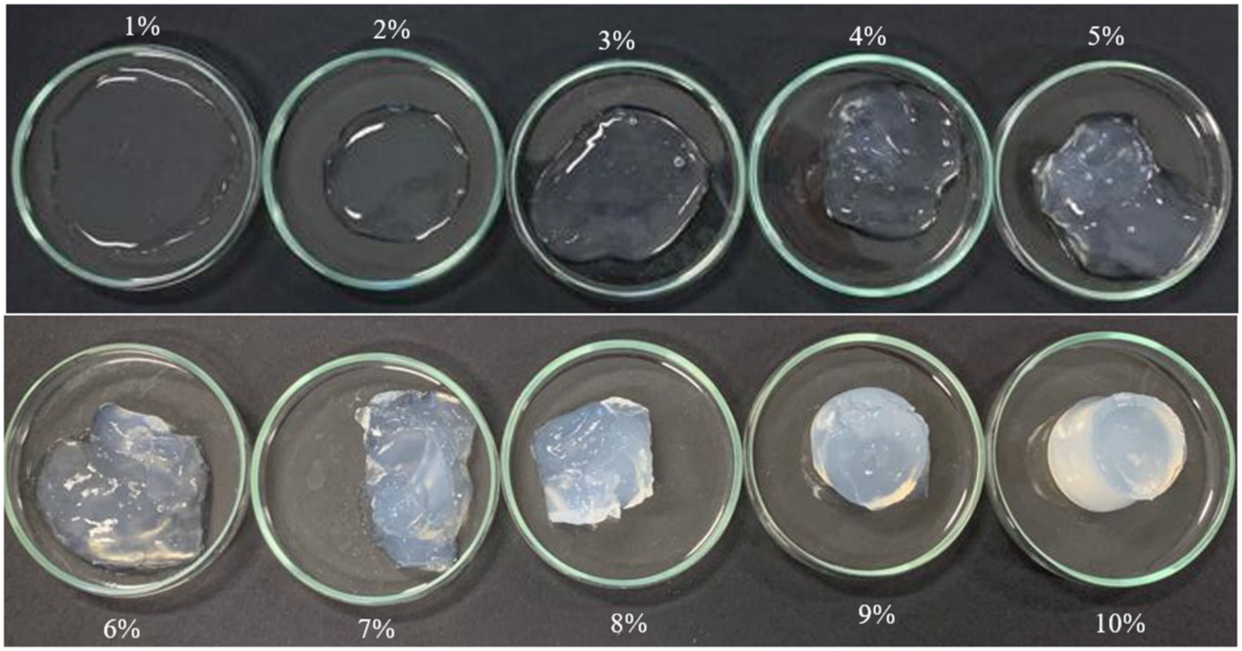

Figure S2. Potato starch hydrogel with various concentrations (i.e., 1% to 10%).

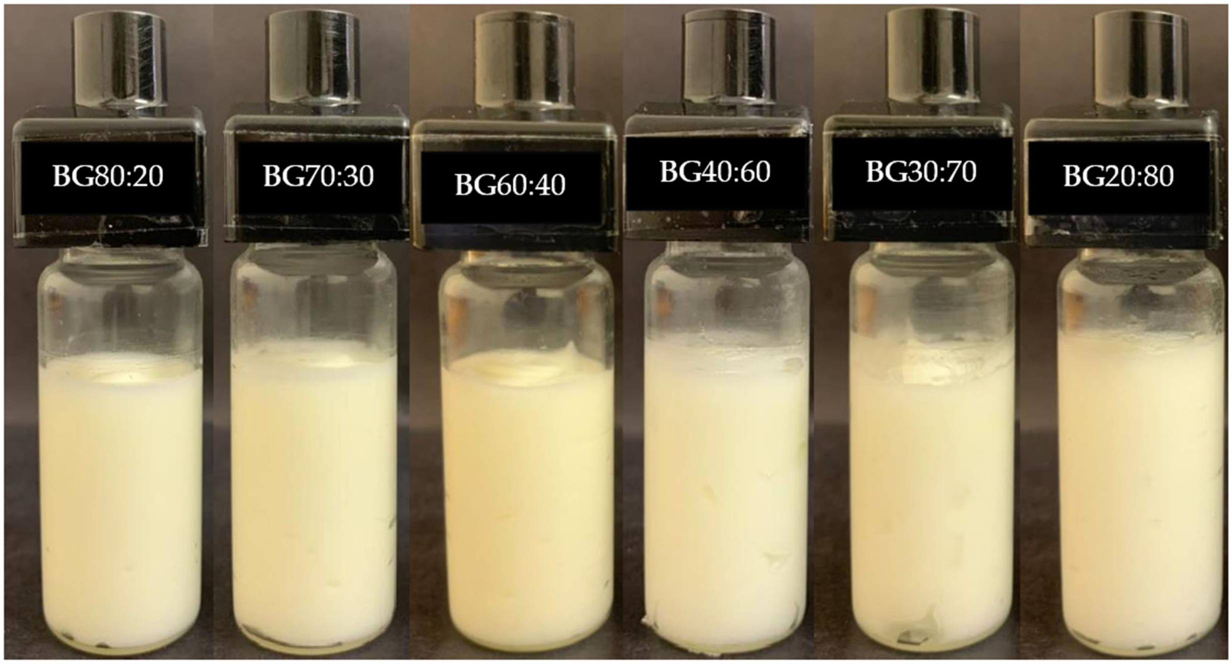

Figure S3. Appearance of bigel (BG) formulations with different oleogel:hydrogel ratios (O:H) after 21 days of storage at 25 °C.
